# Impact of vitamin D on cognitive functions in healthy individuals: A systematic review in randomized controlled clinical trials

**DOI:** 10.3389/fpsyg.2022.987203

**Published:** 2022-11-29

**Authors:** Ana Beatriz Januário da Silva, Waleska Maria Almeida Barros, Mayara Luclécia da Silva, José Maurício Lucas Silva, Ana Patrícia da Silva Souza, Karollainy Gomes da Silva, Matheus Santos de Sousa Fernandes, Antonietta Cláudia Barbosa da Fonseca Carneiro, Ana Elisa Toscano, Cláudia Jacques Lagranha

**Affiliations:** ^1^Programa de Pós-graduação em Neuropsiquiatria e Ciências do Comportamento, Centro de Ciências da Saúde, Universidade Federal de Pernambuco, Recife, PE, Brazil; ^2^Centro Integrado de Tecnologias em Neurociência (CITENC), Centro Universitário Osman Lins (UNIFACOL), Vitória de Santo Antão, PE, Brazil; ^3^Programa de Pós-graduação Multicêntrico em Ciências Fisiológicas, Centro Acadêmico de Vitória, Universidade Federal de Pernambuco, Vitória de Santo Antão, Brazil; ^4^Centro Acadêmico de Vitória, Universidade Federal de Pernambuco, Vitória de Santo Antão, Brazil; ^5^Laboratorio de Bioquimica Geral, Molecular e do Exercicio–Universidade Federal de Pernambuco, Centro Acadêmico de Vitória (CAV)—UFPE, Vitória de Santo Antão, PE, Brazil

**Keywords:** calcitriol, cognition, vitamin D deficiency, cognition function, child

## Abstract

Various functions in the central nervous system, such as growth, development, and cognition can be influenced by vitamins and minerals, which are capable of helping to maintain brain health and function throughout life. Cognition is understood as the aspects related to knowledge, learning, and understanding, as well as the ability to develop these functions. A possible association between low levels of vit D and deficit in the performance of cognitive functions in healthy humans or with some pathological condition is discussed. Because of this, the present systematic review analyzed only randomized clinical trials carried out in healthy non-athlete adults about intellectual and/or mental processes involving cognitive functions to identify whether these individuals with different levels of vit D are capable of interfering with the performance of the cognitive function. To do so, we adopted the PRISMA method criteria and registered it in the PROSPERO database. The search was performed in PubMed (MEDLINE), PsycINFO, Science Direct, Scopus, and Web of Science databases, 2,167 records were identified. The 5 most frequent cognitive domains in the selected studies were: processing speed, attention, verbal learning/memory, executive function, and general cognitive functions. We found that there are positive changes in the following domains: verbal memory and verbal working memory, learning memory, attention, executive function, and also cognitive function in general. We highlight the following suggestions for improvements that vitamin D supplementation may promote in the cognitive domains of healthy adults: a) low doses between 400 and 600 IU/d seem to be more effective when compared to doses between 2,400 and 5,000 IU/d and b) food fortification and enrichment with vit D, need further studies, as they seem to be more or as effective as synthetic supplementation. We evident that there is a need for trials that evaluate the control of vit D levels for healthy adult individuals is important, as they have the potential to minimize health problems, especially those involved in the reduction of cognitive abilities. Thus, the development of more clinical trials to obtain satisfactory answers on this topic needs to be encouraged.

**Systematic review registration:**

https://www.crd.york.ac.uk/PROSPERO/, identifier: CRD42021262413.

## Introduction

Various functions in the central nervous system, such as growth, development, and cognition can be influenced by vitamins and minerals, which are capable of helping to maintain brain health and function throughout life (Rutjes et al., 2018). Vitamin D (vit D) has multiple biological targets mediated by the Vitamin D Receptor (VDR). This vitamin acts on skeletal muscle (Bozsodi et al., 2016), cardiovascular function (Carson et al., 2015), immune cells (Slominski et al., 2022), and also in neurons, and glial cells. In the latter, this vitamin can to regulate neurotransmission, neuroprotection, neuroimmunomodulation, and brain processes, such as the control of calcium homeostasis in hippocampal neurons (Annweiler et al., 2010).

Cognition is understood as the aspects related to knowledge, learning, and understanding, as well as the ability to develop these functions. It consists of the set of skills and cognitive domains that act in synergy for great development and maintenance of intellectual, communicative, and social improvements (Nunes et al., 2021). Some authors point out that vit D is associated with several improvements in clinical health conditions. Its metabolites, for example, have been shown to reduce cancer stem cells in breast and thyroid cancers (Srivastava et al., 2022). Furthermore, supplementation with this vitamin can reduce biomarkers related to beta-amyloid (Aβ) in patients with dementia (Jia et al., 2019).

In addition, a possible association between low levels of vit D and deficit in the performance of cognitive functions in healthy humans or with some pathological condition is discussed (Pettersen, 2017; Beauchet et al., 2019; Fashanu et al., 2019; Eymundsdottir et al., 2020; Xiong and Xue, 2022; Zhang et al., 2022). Clinical trials that verified the effect of vit D supplementation on cognitive functions show that there is a better response to supplementation in individuals who already have some impairment or deficiency of the vitamin (Rossom et al., 2012; Kazem et al., 2014; Hu et al., 2018; Castle et al., 2020; Zajac et al., 2020). Although studies report an association between vit D status and cognitive functions, the potential benefits that this vitamin can promote in terms of cognition are still inconclusive (Gil Martínez et al., 2022).

Although systematic reviews with and without meta-analysis (van der Schaft et al., 2013; Maddock et al., 2017; Rutjes et al., 2018) contribute to a better understanding of the subject, they bring together models with different methodological designs or use only observational studies, which seems to increase conflicting factors. Because of this, the present systematic review intends to gather and analyze only randomized clinical trials carried out in healthy non-athlete adults about intellectual and/or mental processes involving cognitive functions to identify whether these individuals with different levels of vit D are capable of interfering with the performance of the cognitive function.

## Materials and methods

**Figure 1:**
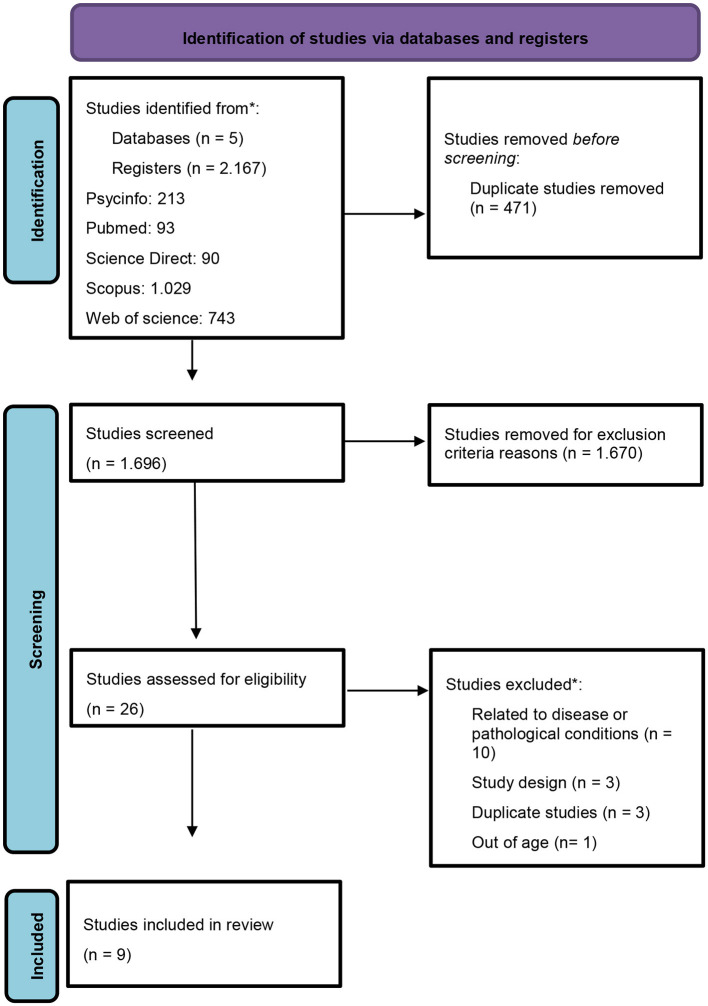
Flowchart of identification of studies via databases. *After reading in full, in the studies selected for eligibility, characteristics that corresponded to the exclusion criteria were observed. Therefore, these studies were not included in this review. From Page et al. (2021).

This systematic review adopted the criteria recommended by the PRISMA method (Preferred Reporting Items for Systematic Reviews and Meta-Analyses), shown in Figure 1. The protocol was registered (CRD42021262413) on 07/22/2021 in the International Prospective Register of Systematic Reviews (PROSPERO) database.

### Search strategy

Searches were performed in the PubMed (MEDLINE), Psycinfo, Science Direct, Scopus, and Web of Science databases with the following search equation: (Vitamin D) AND (((Cognition) OR (Cognitive Function)) OR (Cognitive Functions)), as well as combinations of these terms.

Concerning the combinations of terms that were used, the authors, to seek results that better represent the objective of the present review, also used the terms in different orders. To better locate clinical trials in the databases, the “filter” was used for eliminating the review studies. It is important to note that not all databases offer this filter. Table 1 presents the search strategy information.

**Table 1:** Search strategy information's.

	**PubMed (MEDLINE)**	**Psycinfo**	**Science Direct**	**Scopus**	**Web of science**
**Search equation**	(Vitamin D) “AND” (((Cognition) “OR” (Cognitive function)) “OR” (Cognitive functions))	Vitamin D “AND” Cognition “OR” Cognitive function “OR” Cognitive functions	(Vitamin D) “AND” (((Cognition) “OR” (Cognitive function)) “OR” (Cognitive functions))	(Vitamin D) “AND” (((Cognition) “OR” (Cognitive function)) “OR” (Cognitive functions))	(Vitamin D) “AND” (((Cognition) “OR” (Cognitive function)) “OR” (Cognitive functions))
**Search results**	1,123	-	159	1,696	1,023
**Applied filter**	“Clinical trial” and “Randomized Controlled Trial”	Methodology “Clinical Trial”	“Research articles”	“Article”	“Article”
**Results after applying the filter**	93	213	90	743	743
**Studies included in review**	6	1	2	6	6

The search strategy followed the steps above, the results for “studies included in review” it was speared by databases, in this way the same article can be present in more than one databases. In the Psycinfo database, the search it was only with filter applying.

### Selection of studies and inclusion and exclusion criteria

Two independent researchers (ABJ and MLS) read all titles by database and abstracts, and if the articles fit the review criteria they were read in full. The presence of a third researcher (WMA) was requested in episodes of disagreement. The studies included in this review used the following criteria: (a) studies that performed analyses of vitamin D levels in adults and older adults; (b) those which analyzed intellectual and/or mental processes involving memory, attention, perception, language, or executive functions; (c) only randomized clinical trials were included. Exclusion criteria for the studies were those that: (i) addressed an association with diseases or pathological conditions; (ii) implemented a pharmacological approach; (iii) used athletes or similar models; or (iv) involved physical training protocols.

The PICOS (Population Intervention Comparator Outcome Study design) strategy (Table 2) was also used in the selection of studies for greater specificity. For this, we selected studies carried out with the healthy adult population who underwent vitamin D supplementation or who had their vitamin status analyzed. Outcomes in cognitive functions were analyzed and only randomized clinical trials were used.

**Table 2:** Description of the PICOS strategy that was used.

	**PICOS strategy**	
**Description**	**Abbreviation**	**Components**
Population	P	Healthy individuals non-athletics
Intervention	I	Vitamin D status analysis/Vitamin D supplementation
Comparison	C	Healthy individuals non-vitamin D supplementation or healthy individuals
Outcomes	O	Cognitive functions
Study design	S	Randomized clinical trial

### Data extraction

ABJ and MLS independently reviewed all selected articles to extract relevant data for the preparation of this review. Conflicts were alleviated between authors or with the involvement of the third reviewer. It is important to point out that the searches in the electronic databases were performed without the aid of any automation software in the database search process. For tabulation and extraction of data referring to the selected studies, Excel^®^ software spreadsheets were used.

### Risk of bias

The Joanna Briggs Institute (JBI) tool (Joanna Briggs Institute, 2014) was used to assess the quality of the included studies, where each study was categorized according to the percentage of positive responses to the questions corresponding to the assessment instrument. As a complementary analysis of the risk of bias, the RevMan 5.3.0 software program was used to detect intervening factors from the 7 judgment criteria provided by the program, which are: Random Sequence Generation, Allocation Concealment, Blinding of participants and personnel, Blinding of outcome assessment, Incomplete outcome data, Selective reporting, and Other bias.

## Results

Based on the inclusion criteria and the sum of all databases used, 2,167 titles of articles were initially analyzed. Of these, 471 were excluded because they were duplicates. After verifying the articles in full and according to the pre-established eligibility criteria, 619 studies were excluded because of associated diseases or pathological conditions, and 399 studies were excluded due to the research design. Thus, nine randomized clinical trials performed with adults were included. The flowchart for selecting the included articles is described in Figure 1 (PRISMA).

**Figure 2:**
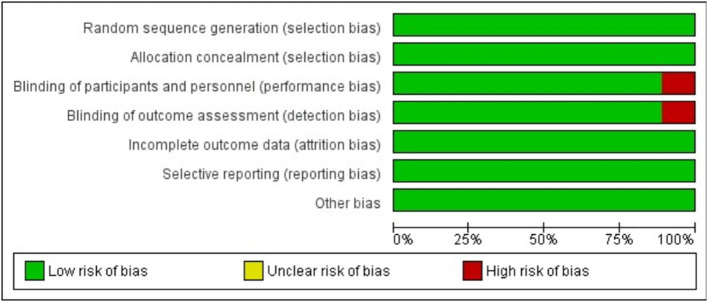
Risk of bias graph: review author's judgements about each risk of bias item presented as percentages across all included studies.

**Figure 3:**
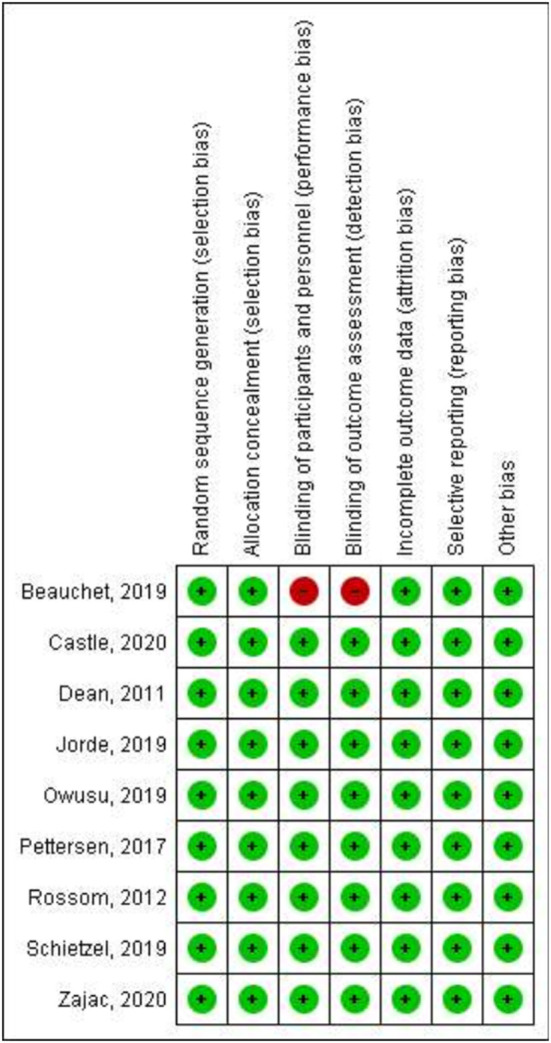
Risk of bias summary: review author's judgements about each risk of bias item for each included study.

Regarding the methodological quality of the studies included in the present review, only one author was not clear about the blinding of the study. Overall, the data had a low risk of bias as shown in Figures 2, 3.

### Study characteristics

Next, three distinct continents were identified regarding the geographic distribution of the studies included in this review (American, Oceania, and Europe). The mean age corresponded to 68.2 ± 16.09 years, and the mean age of the study participants was taken into account. The range of duration of interventions was from 6 to 156.42 weeks. The characteristics of the included studies are presented in Table 3.

**Table 3:** Information on authors, countries and sample characteristics of included studies.

**References**	**Parents**	**Number of Participants**	**Sample Size/Age**	**Gender**	
				**Male**	**Female**
Pettersen (2017)	Canada	142	*n =* 82 high dose group: 56.7 ± 11.7 years low dose group: 52.6 ± 13.4 years	high dose group: 31% low dose group: 35%	high dose group: 69% low dose: 65%
Jorde et al. (2019)	Norway	639	*n =* 394 51.8 years	206	188
Dean et al. (2011)	Australia	139	*n =* 128 21.8 ± 2.9 years	55	73
Rossom et al. (2012)	U.S	4,143	4122 71 years old	0	4,122
Schietzel et al. (2019)	Switzerland	273	226 70.3 ± 6.4 years	127	146
Zajac et al. (2020)	Australia	436	370 69.7 ± 6.6 years	181	189
Beauchet et al. (2019)	France	241	40 71.2 ± 4.4 years	0	40
Castle et al. (2020)	U.S	138	42 58 ± 6 years	0	42
Owusu et al. (2019)	U.S	260	184 68.2 years	0	184

n, sample size.

The research analyzed cognitive domains related to memory, visual abilities, verbal skills, motor skills, temporal/sensory aspects, executive function, logical reasoning, cognitive flexibility, and cognitive function in general. However, due to the range of cognitive domains assessed, those with the highest frequency in the study included: verbal learning/memory, executive function, attention, processing speed, and general cognitive functions.

Regarding vit D, only two studies presented an average of 25(OH)D levels classified as adequate (Dean et al., 2011; Zajac et al., 2020), 4 studies (Rossom et al., 2012; Pettersen, 2017; Owusu et al., 2019; Castle et al., 2020) presented an insufficient classification, and vit D levels were deficient in 3 studies (Beauchet et al., 2019; Jorde et al., 2019; Schietzel et al., 2019) in the initial phase. Importantly, not all studies measured serum vit D levels in their participants before starting a trial intervention, only two trials used this methodology (Jorde et al., 2019; Castle et al., 2020). The specifics of clinical trials are included in Table 4.

**Table 4:** Information on the interventions and variables analyzed.

**References**	**Supplementation time**	**Rating for the 25OHD level at the start of the intervention**	**Administration route/vit D dose**	**Cognitive domains analyzed**
Pettersen (2017)	18 weeks	Insufficient	Oral 1 tablet 400 IU/d 4 pcs 1,000 IU/d	Processing speed; Executive function; Verbal learning/memory.
Jorde et al. (2019)	4 months	Deficient	Oral 20,000 IU/s	Short-term verbal and visual memory; Attention.
Dean et al. (2011)	6 weeks	Adequate	Oral 5,000 IU/d	Executive function.
Rossom et al. (2012)	84 months	Deficient/Insufficient	Oral 400 IU/d	General cognitive function; Attention and working memory; Verbal memory.
Schietzel et al. (2019)	24 months	Deficient	Oral 2,000 IU/d 800 IU/d	Global Cognition; Executive functions; Learning/memory.
Zajac et al. (2020)	6 months	Adequate	Oral (pill or diet) 600 IU/d	Processing speed; Reaction/attention time; verbal and spatial working memory; Overall memory quality.
Beauchet et al. (2019)	3 months	Deficient	Oral Fortified yogurt with 200 IU/2x d	Attention; Processing speed; verbal memory; Attention; Executive function.
Castle et al. (2020)	12 months	Insufficient	Oral 600 IU/d 2,000 IU/d 4,000 IU/d	Executive functioning; Learning/memory; Attention; General cognitive ability.
Owusu et al. (2019)	36 months	Insufficient	Oral 2,400 IU/d 3,600 IU/d 4,800 IU/d	General cognitive function.

IU/d, International Unit per day; IU/s, International Unit per week; Vit D, Vitamin D.

### Vitamin D and cognitive function

Taking into account the 5 most frequent cognitive domains in the studies in this review, the processing speed, attention, verbal/memory learning, executive function, and general cognitive functions and the relationship and/or effect of their modifications in face of different serum vit D dosages were analyzed. The dosages used in the studies ranged from 400 to 4,800 IU/d. Differences were also observed in the type of administration of vit D in two studies (Beauchet et al., 2019; Zajac et al., 2020), which used yogurt and fortified mushrooms containing this vitamin.

Vit D supplementation showed efficacy in increasing 25(OH)D concentrations. However, in the study by Zajac et al. (2020), even using a dosage of 600 IU/d, there were a reduction in 25(OH)D concentration levels during his trial, which lasted a period of 24 weeks. Vit D supplementation regarding cognitive function mainly contributed to improvement in domains related to verbal memory and general cognitive function. The summary of the analyses carried out in this review is organized in Table 5, with the following information: names of the authors, methods for measuring vit D, and the results of the clinical trials included.

**Table 5:** Description of the methods used to analyze the variables and summary of vitamin D outcomes in the cognitive domains.

**References**	**Measurement**		**Results of vitamin D on cognitive domains**
	**Vitamin D**	**Cognitive domains**	
Pettersen (2017)	High performance liquid chromatography mass spectrometry.	SDTM; VF; VRM; OTS.	For 25(OH)D, the high dose (HD) and low dose (LD) groups showed ↑. HD from 63.5 nmol/L to 130 nmol/L (*p =* 0.0001) and BD from 25.4 nmol/L to 85.9 nmol/L (*p =* 0.0001). Δ significantly higher in the HD group compared to the LD (*p =* 0.0001). In the cognitive domains, the LD group compared to the HD group significantly improved in the verbal memory component (*p =* 0.018 and *d =* 0.39). In the subgroup of vit D insufficiency at baseline, the LD group compared to the HD, showed a trend of improvement in the verbal memory component (*p =* 0.054 and *d =* 0.39), but without significance (*p =* 0.09 and *d =* 0.47).
Jorde et al. (2019)	Liquid chromatography – mass spectrometry in tadem.	Short term verbal and visual memory: Verbal recall; The Digit Symbol-Coding Test.	For 25(OH)D, the intervention group showed ↑ significantly from 32.8 ± 11.2 nmol/L to 88.9 ± 19.1 nmol/L, Δ 56.1 ± 22.2 *p =* 0.001. The placebo showed ↓ from 35.3 ± 13.8 nmol/L to 30.8 ± 94 nmol/L, Δ−4.5 ± 13.1. There was no significant difference between groups in Δ on any of the cognitive tests.
Dean et al. (2011)	Tandem mass spectrometry.	Stop signal task.	For 25OHD, the intervention group had a ↑ mean of 76.2 nmol/L to 98.0 nmol/L. There was no difference in the placebo group. There was no change in any of the outcome measures.
Rossom et al. (2012)	Chemilluminescent immunoassay.	Modified MMSE; Digit Span Forward and Backward Test; California Verbal Learning Test.	Baseline 25(OH)D levels did not differ between the treatment and placebo groups (respectively: 20.0 ± 8.8 ng/mL and 19.2 ± 8.4 ng/mL; *p =* 0.36). There was no significant difference in domain-specific cognitive scores between groups.
Schietzel et al. (2019)	Ultra-Performance Liquid Chromatography/Mass Spectrometry.	MMSE; Trail making test B, Stroop Word Color Interference, Digit Span Forwards and Backwards, VF, 5-point Test of Design Fluency; Rey-15 item Test, Rey Auditory Verbal Learning Test, and The Rey Visual design Learning Test.	The 2000 IU/d group had higher 25(OH)D concentrations (Δ7.6 ng/mL with *p* < 0.0001) and ↓ scores for MMSE compared to the 800 IU/d group (27.8 vs. 28.1 with *p =* 0.05). There was no significant change over the 24 months in the 2,000 (*p =* 0.17) and 800 (*p =* 0.90) groups. Likewise, the other outcomes did not differ.
Zajac et al. (2020)	High performance liquid chromatography and tandem mass spectroscopy.	C-CAB; MMSE	Mean 25(OH)D levels ↓ over the 24 weeks (p ≤ 0.01). Interaction effect of the placebo group vs. the group that received vit D2, pointed out that this one showed decline – negative and + slow (*p* = 0.47). General weather effects improved performance in all domains except memory quality. There was an interaction effect only on verbal working memory (*p =* 0.04).
			25(OH)D did not differ between groups (*p =* 0.221) at the start of the intervention. At the end, the intervention group (IG) showed ↑ compared to the control (*p* ≤ 0.001).
Beauchet et al. (2019)	radioimmunoassay	MMSE; Trail Making Test parts A and B; Direct and Indirect Digit Span Score; Stroop Test.	The IG obtained > MMSE score compared to the control 28.5 ± 0.9 vs. 27.1 ± 1.9 (*p =* 0.010). There was a significant Δ intergroup coefficient of variation in the MMSE
			(*p =* 0.022), with a score of ↑ in the GI. The IG performance ↓ in the TMTB throughout the intervention (106 ± 32.1 vs. 88.8 ± 25.2 with p = 0.035).
Castle et al. (2020)	radioimmunoassay	SOC and IED; PAL; RTI; CANTAB and National Adult Reading Test.	No participant had 25(OH)D level <20ng/mL after the intervention and all groups (600, 2,000, and 4,000) ↑~14%, 32% and 50% (p < 0.01). The 2000 UI/d group presented ↑ performance in relation to the others in the PAL test (p < 0.05). The 600 vs. 4,000 IU/d group obtained ↑ result in the IED (< 0.05) and in the RTI (*p < * 0.01). The highest vit D groups had ↓ result in the simple and complex RTI compared to 600 IU/d (*p < *0.01).
Owusu et al. (2019)	Liquid chromatography and quantitative mass spectrometry	MMSE.	MMSE scores increased in both groups (*p =* < 0.001), however the difference between them was not statistically significant.

SDTM, Symbol Digit Modalities Test; VF, Phonemic (verbal) Fluency; VRM, Verbal Recognition Memory; OTS, One-touch Stockings of Cambridge; Δ, illustrative representation to indicate difference; MMSE, Mini-mental State Examination; UI/d, International Unit per day; C-CAB, Cognitive assessment battery CSIRO; TMTB, Trail Making Test part B; SOC, Stocking of Cambridge; IED, Intra/extra-bidimensional Set Shift; PAL, Paired Associates Learning; RTI, Reaction time; CANTAB, Cambridge Neurological Test Automated Battery; “~”, illustrative representation to indicate approximate value.

This review gathered information on 5,588 participants and it was observed that the most used test in the studies to verify the general cognitive function was the Mini-mental state examination test (Beauchet et al., 2019; Owusu et al., 2019; Schietzel et al., 2019; Zajac et al., 2020). The collective of authors Rossom et al. (2012) used a modified version of this test. Some studies (Rossom et al., 2012; Beauchet et al., 2019; Castle et al., 2020; Zajac et al., 2020) used other test batteries, such as the Cambridge Neurological Test Automated Battery (CANTAB), Frontal Assessment Battery (FAB), CSIRO Cognitive Assessment Battery (C-CAB), and The WHISCA cognitive battery (WHI Study Cognitive Aging—WHISCA). Furthermore, isolated tests were also carried out to analyze specific abilities, such as the Digit Span Forward and Backward test, which is available to verify aspects of attention and memory, and also the Stroop test, which analyses executive functions.

Although some of the studies found no effect on the intervention performed with vit D supplementation, others identified a non-significant effect or interaction or positive changes in verbal and non-verbal memory, cognitive function status, executive functions, and attention.

## Discussion

Overall, this review gathered valuable information regarding the effect of vit D supplementation on cognitive functions in healthy individuals. It was found that there are positive changes in the domains of verbal memory and verbal working memory, learning memory, attention, executive function, and also general cognitive function, especially as assessed through the MMSE test. Although most studies resulted in positive cognitive function, a considerable number of studies provided adverse effects, both in the reduction in 25(OH)D concentrations after vit D supplementation (Zajac et al., 2020) and in the worsening of performance in the domains of attention and processing speed (Beauchet et al., 2019).

### Geographic considerations

This synthesis gathers information about the status of vit D and its impact on cognitive function in three continents: North America, Oceania, and Europe. In the case of vit D, it is important to take into account some geographic characteristics, since vit D can photochemically synthesized from ultraviolet (UV) rays. Furthermore, we draw attention to a geographic variable that influences serum vit D levels: latitude.

Latitude and UV radiation are related to vit D levels such that latitudes between 40° parallel North and South offer greater sun exposure, while there is relatively less sunlight available at higher latitudes. About 15% of the world's population lives in higher latitudes and consequently has its ability to convert pre-vitamin D3 reduced, resulting in dependence on the diet to obtain vit D (Ghareghani et al., 2018). Furthermore, UV radiation is capable of being attenuated by melanin through the process of heat dissipation (Jarrett and Scragg, 2020). Thus, it appears that individuals with greater amounts of melanin pigment, with darker skin color, have a reduced capacity to synthesize vit D in the skin (Hedlund et al., 2020).

Thus, all the countries involved in the studies present in this systematic review (Dean et al., 2011; Rossom et al., 2012; Pettersen, 2017; Beauchet et al., 2019; Jorde et al., 2019; Owusu et al., 2019; Schietzel et al., 2019; Castle et al., 2020; Zajac et al., 2020) are in agreement with this information regarding the aforementioned latitude-Vit D relationship when the values before the interventions are observed. In the studies by Dean et al. (2011) and Zajac et al. (2020), the means of 25(OH)D at baseline were 76.6 and 74.4 nmol/L (respectively), which correspond to the proper classification according to the International Osteoporosis Foundation (IOF). These studies took place in Australia and the result is contrary to the information in the Global Overview carried out by van Schoor and Lips (2017). These authors point out that the prevalence of vit D deficiency in the Oceania continent is around 20–50% and still present seven studies carried out in Australia in adults who obtained values below the appropriate classification in their Global Overview.

### Characteristics of participants

#### Brief relationship between age–vitamin D–cognitive function

Overall, the present review presents information regarding 5,628 participants with a mean age of 69.6 years (based on the mean ages provided in the studies), involving individuals from 21.8 ± 2.9 years to 71.2 ± 4.4 years. Due to the various functions that vit D is capable of performing in the human body, its maintenance at adequate levels is essential throughout life. It is important to emphasize the reduction in skin thickness and synthesis capacity during aging, and thus it is common that vit D levels are not adequate at this stage of life (MacLaughlin and Holick, 1985; Holick et al., 1989; Need et al., 1993). As with this information, the authors of this synthesis observed that the mean age of the participants in one of the studies that presented adequate levels of vit D before the interventions was 21.8 ± 2.9 years (Dean et al., 2011). However, participants in another study with the same classification for vit D levels had a mean age of 69.7 ± 6.6 years (Zajac et al., 2020).

The decline in cognitive functions, as well as changes in brain architecture (Lacreuse et al., 2020), such as the reduction in prefrontal white matter volume (Salat et al., 1999; O'Sullivan et al., 2001), are points that are well discussed in the literature regarding the main characteristics of the aging process. In addition, there are other factors capable of causing changes in cognition in healthy people, both in terms of structure and functions, for example, diet (McEvoy et al., 2019; Milte et al., 2019), sleep quality (Dzierzewski et al., 2018), and physical training (Zheng et al., 2019; Raichlen et al., 2020). Thus, it is highlighted that vitamins and minerals can help in the development and maintenance of brain health regardless of age (Rutjes et al., 2018).

The mean age in the randomized clinical trials included in this review which showed some improvement in cognitive function (Pettersen, 2017; Beauchet et al., 2019; Owusu et al., 2019; Schietzel et al., 2019; Castle et al., 2020; Zajac et al., 2020) ranged between 56.7 and 71 years. In addition, the dosages used corresponded to values between 400 and 2,000 UI/d; according to the IOF recommendations, the daily intake of vit D for these age groups is between 800 and 1,000 UI/d. Therefore, doses higher than recommended were used. On the other hand, the age groups in clinical trials which did not report changes in cognitive function (Dean et al., 2011; Rossom et al., 2012; Jorde et al., 2019; Owusu et al., 2019) did not show proximity, with the mean varying between 21.8 and 71 years. Although these clinical trials do not show a positive relationship between vit D supplementation and cognitive function, reviews and meta-analyses show that vitamin deficiency is associated with cognitive difficulties in healthy adults (Balion et al., 2012; Etgen et al., 2012; Annweiler et al., 2013).

In light of this scenario, there are may be some lack of consistency in the findings about vit D and cognitive function, and there are still few clinical trials carried out in the population of young adults. Furthermore, other clinical trials must be carried out to verify the influence of the age group under the effect of vit D supplementation on cognitive functions so that the best strategy for each age group can be consolidated.

#### Considerations regarding pre-intervention vitamin D levels

Vit D is a pre-hormone that can be synthesized by the skin or obtained through the diet, which facilitates its levels to be modulated by the lifestyle and environment in which the individual is inserted. According to the guideline by Pludowski et al. (2018), supplementation of this vitamin is one of the items capable of improving the general health of human beings with the best cost-benefit ratio. It is important to emphasize that the objective of vit D supplementation is to reach and maintain the optimal concentrations of 25(OH)D. Therefore, several studies have used vit D supplementation as a tool for several purposes, such as athletic performance (Dahlquist et al., 2015; Neal et al., 2015; Owens et al., 2015; Chiang et al., 2017; Ksiazek et al., 2019), reduced risk of falls (Annweiler et al., 2010; Bikle, 2014; Halfon et al., 2015), effect on muscle strength/function (Carson et al., 2015; Ebid et al., 2017; Dzik and Kaczor, 2019; da Silva et al., 2021) and cognitive function (Chhetri et al., 2018; Hu et al., 2018; Mayne and Burne, 2019).

When starting any supplementation, an individual in a healthy state who will receive such intervention will normally present an initial value for the parameter under analysis. Thus, in the case of vit D supplementation, the parameter evaluated is the level of 25(OH)D, the main form in which this vitamin is circulating in the human body and is used to clinically monitor the status of this vitamin (Holick, 2005). Therefore, in this review, it was observed that depending on the initial status of the 25(OH)D level presented by the clinical trial volunteer, they would possibly be exposed to two outcomes to the supplementation proposed in the intervention: (a) correction of vit D levels; or (b) improvement in serum vit D status, testing possible doses capable of causing effects on cognition.

The studies by Jorde et al. (2019) and Castle et al. (2020) included in this review described a type of leveling of volunteers participating in the interventions in their methods. Respectively, five capsules of a previous loading dose of 20,000 IU/s were administered during the 4 months of the trial. In this setting, the outcome found was a negative relationship between vit D supplementation and cognitive function. On the other hand, a pre-intervention period of 1 month was defined to level the volunteers' vit D status, and despite the need for other methodological observations, the outcome was positive for the effect of vit D supplementation in the learning/cognitive, memory, executive function and attention domains under the administration of different dosages.

### Intervention models

#### Fortification vs. vitamin D supplementation

Recommendations for daily vit D intake are still conflicting. The National Academy of Medicine (NAM) recommends 600 UI/d for adults between 1–59 and 60–70 years, and 800 IU/d for people aged 71+ years. The IOF did not evaluate recommendations for individuals between 1 and 59 years, but a dose of 800 to 1,000 IU/d is recommended for the group aged over 60 years (International Osteoporosis Foundation, 2021). In addition, the Endocrine Society recommends taking 600 IU/d for individuals at risk of vit D deficiency aged 19–50 years, and 600 IU/d for 50–70 and 70+ (Holick et al., 2011). Furthermore, vit D replacement in adults is dependent on the serum concentration of 25(OH)D. Thus, 50,000 IU/1x per week or 800 IU/d is recommended for <10 ng/mL (<25 nmol/L), while the recommendation for 10–20 ng/mL (25–49 nmol/L) is 800–1,000 IU/d, and finally, 600–800 IU/d is recommended for 20–30 ng/mL (50–74 nmol/L) (Chang and Lee, 2019).

It is not only possible to seek adequacy of vit D levels through supplementation, but also the food fortification technique. There are two ways to carry out the fortification process: (a) mass fortification and (b) fortification point of use, which, respectively, correspond to the addition of micronutrients to foods that are commonly consumed and the addition of single-dose vitamin and mineral packets in the form of powder sprinkled on ready-to-eat food (Das et al., 2019).

In this sense, mass fortification of foods with vit D is carried out by some countries such as the United States, Canada, and Finland in foods such as milk, margarine, yogurt, orange juice, bread, and cereals (Pilz et al., 2018). Vit D is also highly present in sun-dried and UV-exposed mushrooms. According to a review carried out by Cardwell et al. (2018), sunlight and regular and pulsed UV lamps can raise vit D2 concentrations to nutritional significance. Thus, different ways to induce the increase in vit D levels were noted in the clinical trials used in this review; the authors Dean et al. (2011), Rossom et al. (2012), Pettersen (2017), Schietzel et al. (2019), Jorde et al. (2019), Owusu et al. (2019), and Castle et al. (2020) used tablet/capsule supplementation in their clinical trials.

On the other hand, in addition to vit D3 capsules, Zajac et al. (2020) used mushrooms with increased vit D2 through exposure to UV rays to assess whether vit D2 and D3 were casually related to cognition and mood in healthy individuals. In this case, doses of 600 IU/d were used, and it is important to note that the processes of lyophilization and crushing of mushrooms were carried out for this administration, followed by exposure to UV-B light. Another alternative used to increase serum vit D levels also found in selected studies was yogurt fortification. Beauchet et al. (2019) used 200 IU of vit D3 and 400 mg of calcium per size of yogurt. In this trial, the hypothesis was that low daily doses of vit D (400 IU/d) associated with 800 g of calcium through fortified yogurt for 3 months would improve cognitive performance and therefore 2 pots of yogurt were administered per day.

In agreement with the data found in the literature regarding the efficacy of food fortification and the use of mushrooms enriched using UVB light, despite the two clinical trials (Beauchet et al., 2019; Zajac et al., 2020) working with different dosages of the 600 IU/400 vitamin UI/d (respectively), both had positive outcomes regarding the influence of vit D on cognitive functions and performance, more precisely processing speed, action/reaction time, verbal and spatial working memory, and general cognitive functions assessed by the MMSE.

### Tests for evaluation of cognitive function

We present in Table 4 the cognitive domains that were evaluated and in Table 5 we present the tests used to assess the cognitive function in question. Given this, it is possible to observe that some studies used isolated tests to verify the desired cognitive function or domain, and other authors used a battery of tests to identify one or more domains. For global assessment of cognitive function, a commonly used test was the MMSE, which is a diagnostic test for dementia. Its use was identified in more than one study present in this review, to apply the score as an evaluation parameter for the effect of vit D supplementation.

In addition to an article using a modified version of the MMSE (Rossom et al., 2012) for global assessment of cognitive function, another (Castle et al., 2020) used the National Adult Reading Test (NART) for such measurement. The NART (Bright et al., 2002) is used to quantify pre-morbid intelligence, that is, when the individual is before the known or suspected onset of a brain disease or dysfunction. However, on that occasion, the effect of vit D supplementation in the domains measured by this test was not quantified, which makes the replication process of the study difficult.

Furthermore, test batteries such as C-CAB, WHISCA, FAB, and CANTAB were also used in the articles selected for this review. Since skills such as non-verbal memory, reaction time, and global cognitive performance showed positive changes after vit D supplementation and most of the aforementioned batteries of tests can measure these skills, the proper use of these skills is evident. On the other hand, what caught our attention was the FAB test battery used by Beauchet et al. (2019). This battery aims to specifically assess the functions of the frontal lobe, being sensitive to its dysfunctions. A systematic review carried out by Hurtado-Pomares et al. (2018), makes important scores about FAB which, although it manages to assess skills involved in executive functioning, is much more sensitive to tracking cognitive dysfunctions.

Given the above, we highlight the importance of the quality of methodological rigor when choosing the tests or battery of tests that are in line with what is objectified. Although the test is capable of measuring a measure of interest, it is necessary to verify its sensitivity to other characteristics, such as different populations or health conditions.

### Vitamin D and cognitive function

Cognitive function is the result of several complex systems involving many local neural circuits (Birle et al., 2021). In addition, it is also understood as intellectual skills that develop knowledge and is subdivided into components such as attention, intelligence, memory, and decision-making (Spiga et al., 2008). In the present review, positive changes were found in some domains: memory, executive function, attention, and also in the MMSE, which is a test used to identify changes in the general state of cognitive function.

Another review that evaluated the effects of vitamin and mineral supplementation on cognitive function of 363 individuals aged 40 years and over had 28 studies and 2 were related to vit D (Rutjes et al., 2018). In the aforementioned review, it was not possible to identify any effect of vit D supplementation on cognitive functions, conflicting with our findings. However, it is important to emphasize that in addition to the few studies that were analyzed, those that were included, according to the authors themselves, presented a high risk of bias.

The review carried out by Annweiler et al. (2009), analyzed data that assessed the association between vit D levels and adult cognitive performance. Five studies were included in this review which, like the present work, identified in part of the studies an association between vit D levels and cognitive function and in another part the absence of such association. It is necessary to point out that in the review by Annweiler et al. (2009), the studies analyzed were observational, thus differing from the present review, which included only randomized clinical trials.

## Highlighting limitations and strengths

Our study has several highlighting strengths. We used the items indicated by PRISMA to systematize our review, using a checklist and for building a flowchart. In addition to this, Cochrane's Review Manager (RevMan) software was also used to assess the methodological quality of the studies selected by the eligibility criteria, ensuring the exposure of biases judiciously.

The limitations presented in the present work are related to the designs of the selected studies. Although they have explained information about the methodology, some of the studies included in the present review used groups with the intervention of high-dose vit D supplementation and control groups also using vit D supplementation, at low doses. This may have corroborated the absence of improvements in cognitive function, as gains from supplementation were not compared against the absence of supplementation.

In addition, some studies used the MMSE. This test is used in the diagnostic evaluation of possible dementia, in clinical trials the MMSE was used to verify the state of cognitive function in general so that improvements in overall cognitive function were considered if the score was increased in pre- and post-intervention measurements. Given the complexity of cognitive functions, the MMSE test becomes limited when it is intended to measure routine cognitive functions.

## Final considerations

Due to the heterogeneity of the studies included in this review, it was not possible to define the duration and adequate dose of vitamin D supplementation to improve cognitive functions. Therefore, the development of public policies on this theme needs to be however encouraged. However, it is suggested that vitamin D supplementation to improve cognitive domains in healthy adults: (a) low doses between 400 and 600 IU/d seem to be more effective when compared to doses between 2400 and 5000 IU/d; (b) food fortification and enrichment with vit D, need additional studies, as they seem to be more or as effective as synthetic supplementation; (c) more clinical trials with young adults are needed, to understand the possible positive changes promoted by supplementation with this vitamin in cognitive functions.

Although cognitive dysfunctions are often associated with low levels of vit D (Hung et al., 2022; Saji Parel et al., 2022; Zhang et al., 2022), the effects of the possible benefits that this vitamin may provide to cognition remain inconclusive. It was evident the need for trials that evaluate, with good methodological quality, the supplementation of vit D in healthy individuals under 60 years of age and its effects on cognitive function.

Evident that there is a need for trials that evaluate the control of vit D levels for healthy adult individuals is important, as they have the potential to minimize health problems, especially those involved in the reduction of cognitive abilities. Thus, the development of more clinical trials to obtain satisfactory answers on this topic needs to be encouraged.

## Data availability statement

The raw data supporting the conclusions of this article will be made available by the authors, without undue reservation.

## Author contributions

AS and WB contributed to research conception, data collection, interpretation of results, and critical review of the article. MS, JS, AS, KS, MdSF, and AC contributed to data analysis and interpretation, drafting, and critical review of the article. AT and CL contributed to data collection and critical review of the article. All authors contributed to the article and approved the submitted version.

## Conflict of interest

The authors declare that the research was conducted in the absence of any commercial or financial relationships that could be construed as a potential conflict of interest.

## Publisher's note

All claims expressed in this article are solely those of the authors and do not necessarily represent those of their affiliated organizations, or those of the publisher, the editors and the reviewers. Any product that may be evaluated in this article, or claim that may be made by its manufacturer, is not guaranteed or endorsed by the publisher.
