# Corrigendum: Impact of vitamin D on cognitive functions in healthy individuals: A systematic review in randomized controlled clinical trials

**DOI:** 10.3389/fpsyg.2023.1150187

**Published:** 2023-02-28

**Authors:** Ana Beatriz Januário da Silva, Waleska Maria Almeida Barros, Mayara Luclécia da Silva, José Maurício Lucas Silva, Ana Patrícia da Silva Souza, Karollainy Gomes da Silva, Matheus Santos de Sousa Fernandes, Antonietta Cláudia Barbosa da Fonseca Carneiro, Ana Elisa Toscano, Cláudia Jacques Lagranha

**Affiliations:** ^1^Programa de Pós-graduação em Neuropsiquiatria e Ciências do Comportamento, Centro de Ciências da Saúde, Universidade Federal de Pernambuco, Recife, PE, Brazil; ^2^Centro Integrado de Tecnologias em Neurociência (CITENC), Centro Universitário Osman Lins (UNIFACOL), Vitória de Santo Antão, PE, Brazil; ^3^Programa de Pós-graduação Multicêntrico em Ciências Fisiológicas, Centro Acadêmico de Vitória, Universidade Federal de Pernambuco, Vitória de Santo Antão, Brazil; ^4^Centro Acadêmico de Vitória, Universidade Federal de Pernambuco, Vitória de Santo Antão, Brazil; ^5^Laboratorio de Bioquimica Geral, Molecular e do Exercicio–Universidade Federal de Pernambuco, Centro Acadêmico de Vitória (CAV)—UFPE, Vitória de Santo Antão, PE, Brazil

**Keywords:** calcitriol, cognition, vitamin D deficiency, cognition function, child

In the published article, there was an error in the author list as published. Author Viviane de Oliveira Nogueira Souza was erroneously included and author Ana Elisa Toscano was erroneously excluded.

The authors apologize for these errors and state that this does not change the scientific conclusions of the article in any way. The original article has been updated.

